# EpiGeoPop: a tool for developing spatially accurate country-level epidemiological models

**DOI:** 10.1038/s41598-025-11999-4

**Published:** 2025-07-22

**Authors:** Lara Herriott, Henriette L. Capel, Isaac Ellmen, Nathan Schofield, Jiayuan Zhu, Ben Lambert, David Gavaghan, Ioana Bouros, Richard Creswell, Kit Gallagher

**Affiliations:** 1 SABS R3 CDT, University of Oxford, Oxford, UK; 2 Mathematical Institute, University of Oxford, Oxford, UK; 3 Department of Computer Science, University of Oxford, Oxford, UK; 4 Department of Statistics, University of Oxford, Oxford, UK

**Keywords:** Infectious diseases, Software

## Abstract

Mathematical models play a crucial role in understanding the spread of infectious disease outbreaks and influencing policy decisions. These models have aided pandemic preparedness by predicting outcomes under hypothetical scenarios and identifying weaknesses in existing frameworks; however, their accuracy, utility, and comparability are being scrutinised. Agent-based models (ABMs) have emerged as a valuable tool, capturing population heterogeneity and spatial effects, particularly when assessing potential intervention strategies. Here we present EpiGeoPop, a user-friendly tool for rapidly preparing spatially accurate population configurations of entire countries. EpiGeoPop helps to address the problem of complex and time-consuming model set-up in ABMs, specifically improving the integration of real-world spatial detail. We subsequently demonstrate the importance of accurate spatial detail in ABM simulations of disease outbreaks using Epiabm, an ABM based on Imperial College London’s CovidSim with improved modularity, documentation and testing. Our simulations present a number of possible applications of ABMs where including spatially accurate data is crucial, highlighting the potential impact of EpiGeoPop in facilitating this process using multiple international data sources.

## Introduction

Mathematical models played a critical role over the course of the COVID-19 pandemic, aiding our understanding of the factors underlying disease spread and, consequently, strongly influencing policy decisions^1,2^. These models are a key tool for pandemic preparedness, allowing the impacts of hypothetical disease scenarios to be predicted^3^. In addition to predicting the temporal evolution of epidemics, estimating the impact of a range of interventions (such as isolating infected cases and closing schools or workplaces) plays a significant role in guiding government policy^4,5^. The CovidSim model^4^, developed by Imperial College London, is one such mathematical model that informed the early pandemic response in the UK and US by predicting the impact of different government interventions^6^. Given the ongoing risk of future pandemics^7^, improving the accuracy and usability of such models is an essential step towards pandemic preparedness.

Agent-based models (ABMs) and differential equation models represent the two major modelling frameworks in epidemiology^8^. ABMs model members of a population individually and thus can natively handle population heterogeneity^9,10^. They allow detailed and informative predictions to be obtained in settings where inter-individual variability in traits, behaviour and interactions is important^10^. In contrast, differential equation models describe the spread of a disease through a system of equations modelling continuous state variables, resulting in a deterministic model that averages individual traits across a population^11,12^. To represent an epidemic, differential equation models contain compartments representing the various stages of disease transmission, such as susceptible, exposed, infected (possibly with multiple degrees of severity^13^), and recovered^13–16^.

Differential equation models can also be adapted to incorporate population heterogeneity through the addition of further compartments such as for age structure^12,14,15^ or within-household infection pathways^15^. However, increasing the number of compartments requires a corresponding increase in the number of equations in the model, which effectively places an upper limit on the amount of detail which can realistically be included within this modelling framework. As a consequence, very few models implement multiple aspects of population heterogeneity simultaneously.

Representations of population heterogeneity are important when assessing the impact of a range of interventions on mitigating the spread of disease, which is key to informing government policy. School closure, for example, can be captured in compartment-based models with age structure by adjusting the parameters governing disease transmission between school-aged children^17^. In contrast, school closure can be implemented in an ABM by preventing agents representing both school-aged children and adult teachers from attending virtual schools^18,19^. Furthermore, additional complexities and knock-on effects of such an intervention, such as decreased workplace visits among parental groups, could also be captured^20^ using the same place-based structure. As such, the ability to mechanistically model the effect of multiple, complex interventions is a major advantage of ABMs over differential equation models. However, including such individual-level detail in these models comes at a cost, either in the time spent sourcing the relevant spatial and demographic data, or through the additional assumptions which must be made^21–23^. On the other hand, the relative simplicity of differential equation models, typically involving many fewer parameters, makes them better placed for addressing other questions, such as those involving inference^24^. In short, the additional ‘real-world’ complexity of ABMs makes them more ‘data hungry’ than differential equation models^25^.

Given their ability to capture substantial population heterogeneity, ABMs are particularly well suited to modelling the effect of spatial heterogeneities on disease spread. While few studies had explicitly considered the impact of population density on the large-scale spread of infectious diseases prior to the COVID-19 pandemic^26,27^, the collection of vast amounts of infection data globally over the past five years has allowed this topic to be explored in greater detail. For example, understanding how the distribution of population density shapes disease spread is crucial for informing containment and mitigation policies and determining the effectiveness of localised restrictions^28,29^. In this way, an understanding of the effects of spatial patterns could improve pandemic preparedness.

Previous tools and models have been developed to streamline the construction of ABMs using real-world data^30,31^. The importance of using spatial data, so that the resulting model populations are defined in terms of their specific geography, has also been highlighted previously^32^. However, to the best of our knowledge, most of the currently available pipelines process data for a select number of countries only and are intrinsically linked to specific epidemiological models. There is therefore a need to develop more flexible approaches to population configuration that are agnostic to the ABM itself and can be easily applied to generate populations for different countries or geographic regions of interest based on real-world data.

In the present work, we begin to address the difficulty of incorporating real-world data into spatially accurate epidemiological ABMs by presenting a user-friendly tool, EpiGeoPop, for easy and rapid configuration of spatially detailed ABM populations. In contrast to previous country-specific tools, EpiGeoPop extracts spatially segregated population data for geographical regions across the globe, which are then compiled into human-readable input files. In addition, EpiGeoPop generates population-specific parameter estimates from published data. EpiGeoPop can be used in conjunction with existing epidemiological models to speed up configuration of realistic populations based on country-specific, real-world data.

We demonstrate the utility of EpiGeoPop, and the importance of incorporating accurate spatial detail into epidemiological models, by running example simulations on a national scale. For this we use the epidemiological model Epiabm, an alternative implementation of CovidSim^4^ which emphasises modularity, documentation, and good software engineering practices^33^. Epiabm and EpiGeoPop were developed as part of the first-year training in software engineering for PhD students in the EPSRC CDT in Sustainable Approaches to Biomedical Science: Responsible and Reproducible Research (SABS:R^3^) CDT at the University of Oxford.

## Methods

Here, we describe EpiGeoPop (our tool for constructing spatially accurate model populations) and discuss our improvements and extensions to Epiabm (the ABM we use for further configuring our model populations and then running epidemic simulations). The original release of Epiabm did not implement pharmaceutical or non-pharmaceutical interventions, and used a simplistic implementation of spatially mediated infections. Our extensions to Epiabm include both forms of intervention and an improved method for weighting spatial infections. Open-source code for both EpiGeoPop and Epiabm are available at https://github.com/SABS-R3-Epidemiology/EpiGeoPop and https://github.com/SABS-R3-Epidemiology/epiabm.

### EpiGeoPop

**Fig. 1:**
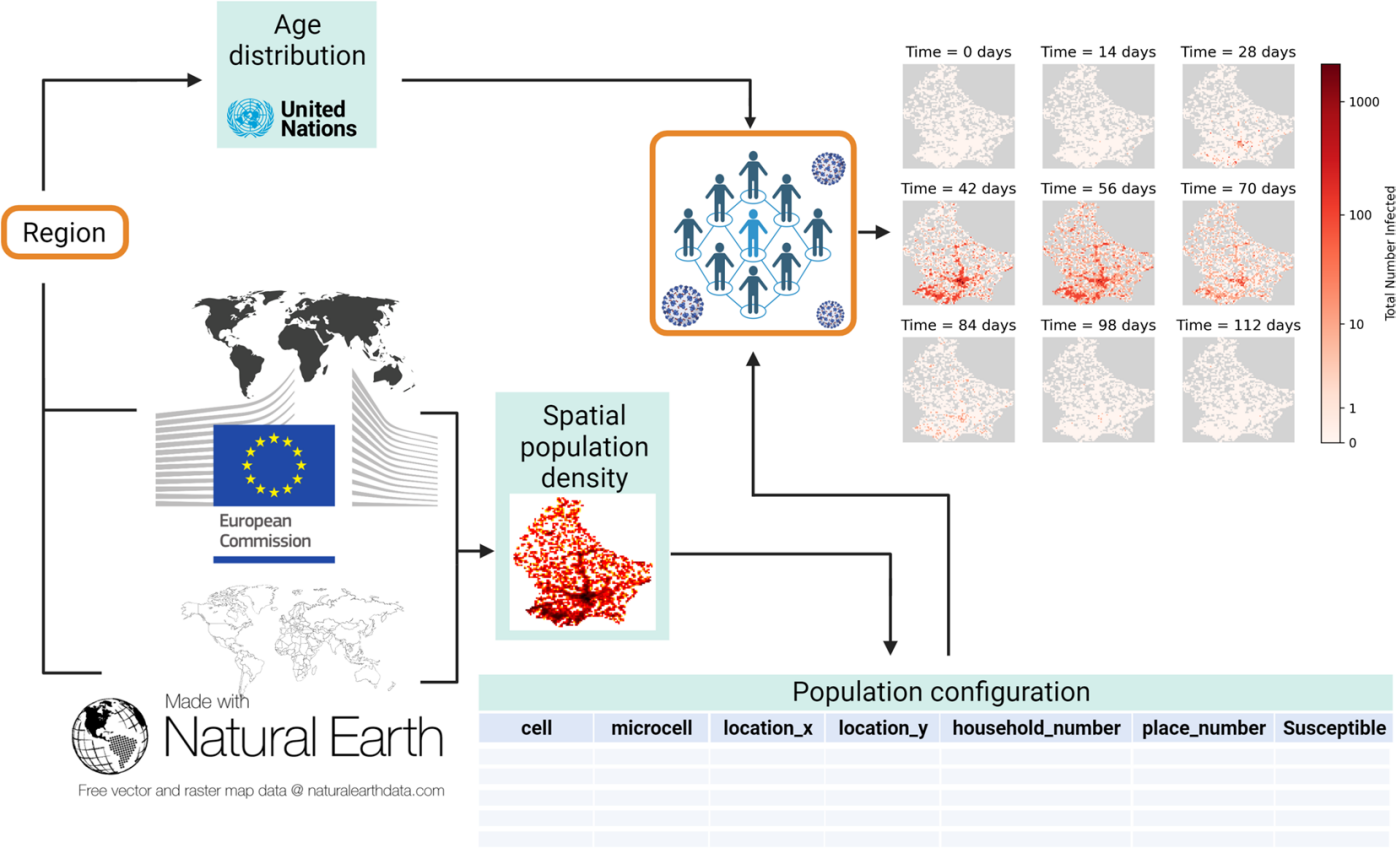
EpiGeoPop workflow. EpiGeoPop uses global population density data to generate population configuration files for any country, province, or city of interest. The configuration file contains one row per ‘microcell’ (the smallest defined spatial region) with columns recording the microcell and its parent ‘cell’, the location (in x and y coordinates) of that parent cell, and the number of households, places, and susceptible individuals in that microcell. In addition, age distributions are generated on a country-level basis. The choices of region and epidemiological model, highlighted in orange, represent the elements of the workflow determined by the user. As long as the overall format of the output file from the model matches that of the input file generated by EpiGeoPop (i.e., columns representing the location’s x- and y-coordinates and the number of individuals at that location), the visualisation module can be used. This allows geographically accurate visual summaries of disease dynamics to be generated in the form of GIFs. Created with BioRender.com.

A pre-requisite for spatially accurate epidemiological modelling is the integration of spatial data into the modelling framework. To simplify these initial stages of model set-up, we develop a software tool named EpiGeoPop (Fig. 1) which constructs a geographically accurate density map from global population density data^34^ and converts this into a human-readable population configuration file. Our tool allows this input file to be generated for any country or region of interest for which the border and population density data are available in the Natural Earth (https://www.naturalearthdata.com/downloads/10m-cultural-vectors/) and European Commission, Joint Research Centre Global Human Settlement Layer^34^ databases, respectively. We use the 2015 version of the population density dataset, which includes regions on the scale of cities, provinces and complete countries. The 2015 data, alongside datasets for other years, can be found at: https://human-settlement.emergency.copernicus.eu/download.php?ds=pop

#### The EpiGeoPop workflow

For ease of use, we present a complete workflow as a user-friendly Snakemake pipeline^35^ implemented in Python. This pipeline reads in the border and population density data described above, and generates a population configuration file that describes the distribution of individuals across a spatial grid with a resolution 30 arc seconds (approximately 1 km^2^). We label these $\sim 1$ km^2^ regions ‘cells’. EpiGeoPop allows these cells to be optionally split further into ‘microcells’, should a smaller resolution be required. In the simulations presented herein, we use a grid size of 3x3 totalling nine microcells per cell, though the specific number of microcells can be specified by the user.

Additionally, users can specify parameters to add a number of places and households to each microcell in the population. Overall, the population configuration output file contains one line per microcell, stating the parent cell of that microcell and its location, as well as the attributes of the microcell, namely the number of households, places, and individuals that are contained within it. This structured hierarchy of people, places and households existing in microcells, which in turn exist within cells, is provided for in EpiGeoPop to ensure compatibility with Epiabm. This may not be applicable depending on the ABM being used, in which case the user can simply opt not to break cells into multiple microcells or to define a number of places and households.

EpiGeoPop also generates country-specific age distributions, taken from the UN 2022 Revision of World Population Prospects^36^. These are processed to represent the proportion of individuals in 5-year age groups from 0-80, with a final group of over 80 year-olds, resulting in an age distribution compatible with Epiabm.

Details on how to execute the workflow, tailored to the user’s specific needs, are provided in the Supplementary Information, Section S1.1.

#### Visualising epidemic spread

We also provide code to generate time-series animations of disease spread across the region of interest (examples of this are provided in Supplementary Videos 1 and 2). This code is run as a single Python script that creates a GIF visualising the infection counts as a heatmap changing over time. To use this script, the output data from the epidemiological simulation should be provided in the format matching that generated by the Snakemake workflow (details are provided at https://github.com/SABS-R3-Epidemiology/EpiGeoPop/#generating-animations).

### Epiabm

We utilise Epiabm (v1.1.0) for all epidemic simulations, extending this software from the previously published version (v1.0.1). Epiabm includes algorithms for configuring age, household, and place structure within the model population. Households and places sit at the ‘microcell’ level, with microcells sitting within larger geographic regions termed ‘cells’ as above. The model uses a compartmental structure to simulate disease spread, with compartments for susceptible, infected (of varying severity), recovered, and dead individuals. Infected individuals are able to infect susceptible individuals through three transmission pathways: place-based, household-based, and spatial-based. Our extended version of Epiabm includes an altered weighting for spatial transmission, and implements a suite of time-limited events, including pharmaceutical and non-pharmaceutical interventions.

#### Improved weighting of spatial transmission

Spatial transmission across cells involves the random selection of one cell for each person a given infector should infect. The infectee is then selected at random from that cell’s population. In both CovidSim and the initial version of Epiabm (v1.0.1), this cell is selected with probability inversely proportional to its distance from the infector’s cell. Although travel between locations is not explicitly modelled in Epiabm, this method of spatial transmission has the effect of considering travel to two locations the same distance apart equally likely, irrespective of their population.

However, to accurately model the impact of heterogenous population density distributions, we adjusted the spatial infection mechanism such that an infectee is more likely to be selected from a higher-density cell than a lower-density cell the same distance away. In effect, this approximates the impact of transport networks with hubs centred on larger towns and cities^37^. The effect of this change is presented in Figs. 3 and 4 of the Results section ‘Population density influences epidemic dynamics’.

#### Non-pharmaceutical interventions

We additionally implemented the full suite of non-pharmaceutical interventions present in CovidSim^4^: case isolation, household quarantine, place closure, and social distancing. In our implementation of these interventions, we closely follow that used in CovidSim. Interventions can be activated at a specific simulation time, or when the total number of cases crosses a specified threshold, capturing some of the flexibility present in CovidSim. Multiple interventions can be active at the same time, and the same intervention can be implemented over different time periods with varying severity. All non-pharmaceutical interventions influence house, place, and spatial infections. When case isolation is active, symptomatic or positive-tested individuals will isolate within their household for a given period (for a description of the testing functionality please see the section ‘Pharmaceutical interventions’). Household quarantine is built upon case isolation and restricts members of the household of the isolated individual to also stay at home for a defined number of days. Place closure allows for the closure of specific place types, such as schools, workplaces, or outdoor spaces, to be simulated. Social distancing reduces the likelihood of house, place, and spatial infections. We allow for two strengths of this reduction, which can be applied to different age groups. Simulations including these interventions have the expected effect of delaying and reducing the peak of infections (Fig. S3)

#### Pharmaceutical interventions

In addition, we implement two pharmaceutical interventions: mass vaccination and infection testing to determine case isolation. Mass vaccination can be prioritised according to individual characteristics, and the protection afforded by vaccination can be parametrised by the user. Infection testing includes two testing streams for which parameters including sensitivity, specificity, testing capacity and type of user can be set. These two streams can therefore be adjusted to represent different testing modalities, such as PCR and lateral flow tests. Testing can then be used as a criterion to select an individual for case isolation instead of using their symptomatic status.

#### International travel

Finally, we develop a simple travel framework to capture international travel. Through this functionality, border closure and isolation of international arrivals (either within an existing household or in separate accommodation) can be activated as an additional intervention.

### Combining EpiGeoPop and Epiabm to configure and run simulations

In the simulations presented herein, we generate population configuration files of real countries using EpiGeoPop, and simulate the spread of disease through these populations using Epiabm. Though we use the terminology of cells and microcells in both EpiGeoPop and Epiabm, the concept of these spatial regions is scale-agnostic in Epiabm, while in EpiGeoPop the resolution of the spatial data used means cells are considered to be $\sim 1$ km^2^ regions, which can optionally be broken down into microcells.

We use the population configuration file output by EpiGeoPop to define the spatial distribution of the population. We then apply an algorithm included in Epiabm to assign ages and households to individuals according to the age and household distributions for the country of interest. The age distribution is also provided by EpiGeoPop, and we take the household distributions from data provided by the United Nations Population Division: https://www.un.org/development/desa/pd/data/household-size-and-composition. We verify this algorithm by comparing the age and household distributions of the configured population after application of the algorithm to the input distributions (Fig. S1).

## Results

We present simulations to demonstrate the importance of including spatial population density detail in epidemiological models. In addition, we investigate how interventions differentially impact different density regions. Taken together, the simulations presented aim to demonstrate possible applications of both EpiGeoPop and Epiabm for investigating epidemic dynamics.

### Population density influences epidemic dynamics

**Fig. 2:**
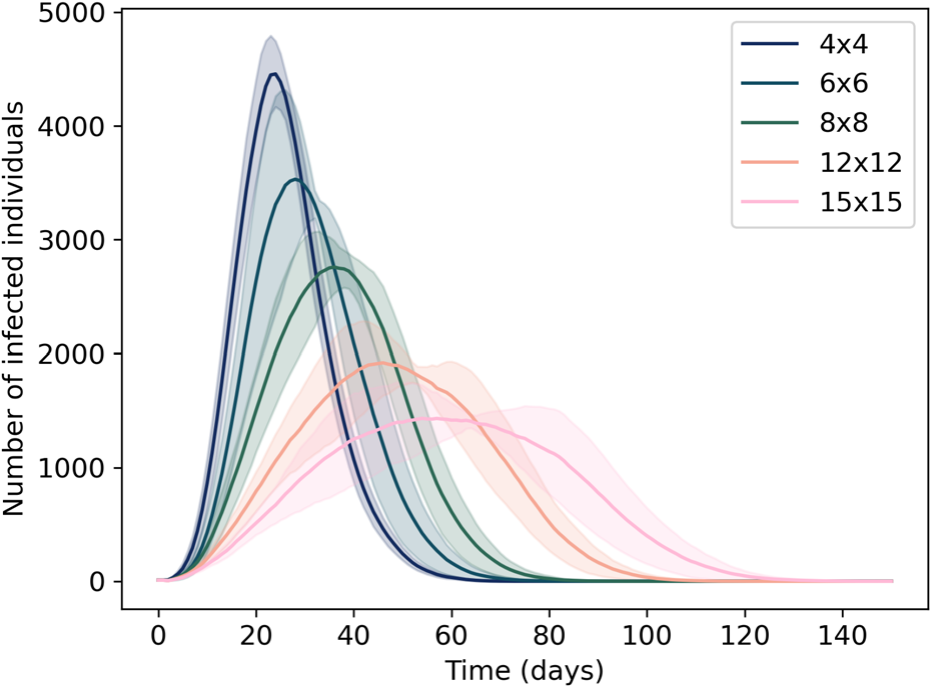
Lower-density populations experience delayed and lower peaks of infections. Populations of 10,000 individuals are spread approximately uniformly over square grids of dimensions 4x4, 6x6, 8x8, 12x12, and 15x15. As such, the 4x4 model population represented the highest density, while the 15x15 is the lowest density. Lines indicate the mean over 10 simulations, while the shaded areas represent the standard deviations.

Simulations for five approximately uniform populations with differing population density reveal a pattern of lower and later peaks in lower-density populations. In our highest-density population (4x4 grid), the number of infections reaches a peak 30 days earlier and at a value more than three times greater than that in the lowest-density population (15x15 grid) (Fig. 2). These results indicate the substantial influence that population density can have on the spread of infections, even when this density is homogeneous. They also motivate consideration of the effects of heterogeneous population densities on disease spread, as exists in real countries.

Using EpiGeoPop, we constructed a spatially accurate model of Luxembourg. We selected Luxembourg for investigative purposes since its population density varies widely across the country, while its population size (569, 335, as of 2015) remains sufficiently small for simulations to be run locally in a reasonable time ($\sim 45$ min). Details of all simulations run for Luxembourg can be found in the Supplementary Information (Section S1.4).

**Fig. 3:**
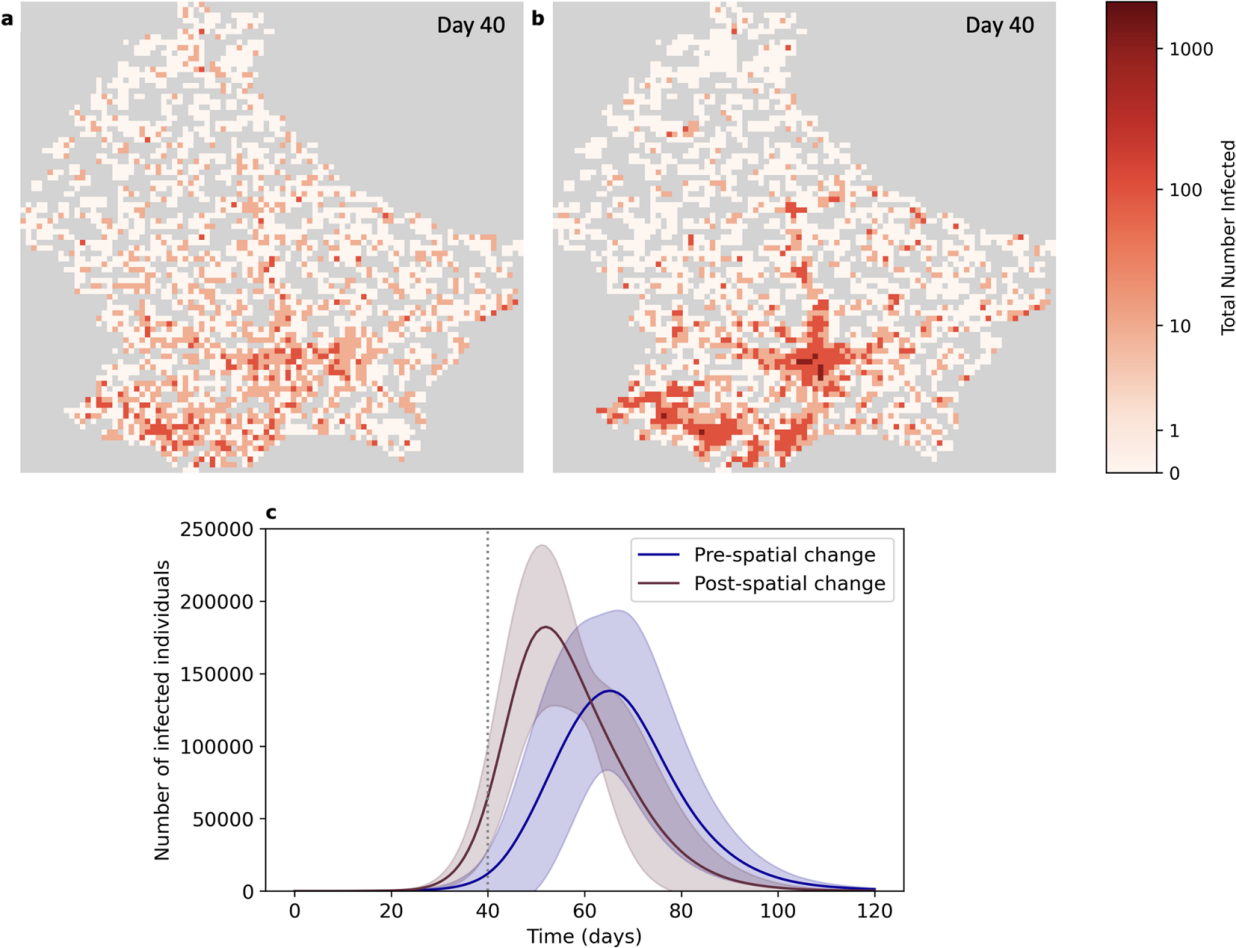
Disease transmission in Luxembourg before and after changing the spatial transmission weightings. **(a)** Snapshot of the distribution of infections at day 40 with spatial infections guided by distance only. **(b)** Snapshot of the distribution of infections at day 40 with spatial infections guided by distance and population. In both snapshots darker colours indicate higher numbers of infections. **(c)** Total number of infections over time before (blue) and after (red) the change to the spatial transmission weightings. Results are shown as the mean ± standard deviation over 10 repetitions of each simulation. The grey dashed line indicates day 40, corresponding to the time of the above snapshots.

**Fig. 4:**
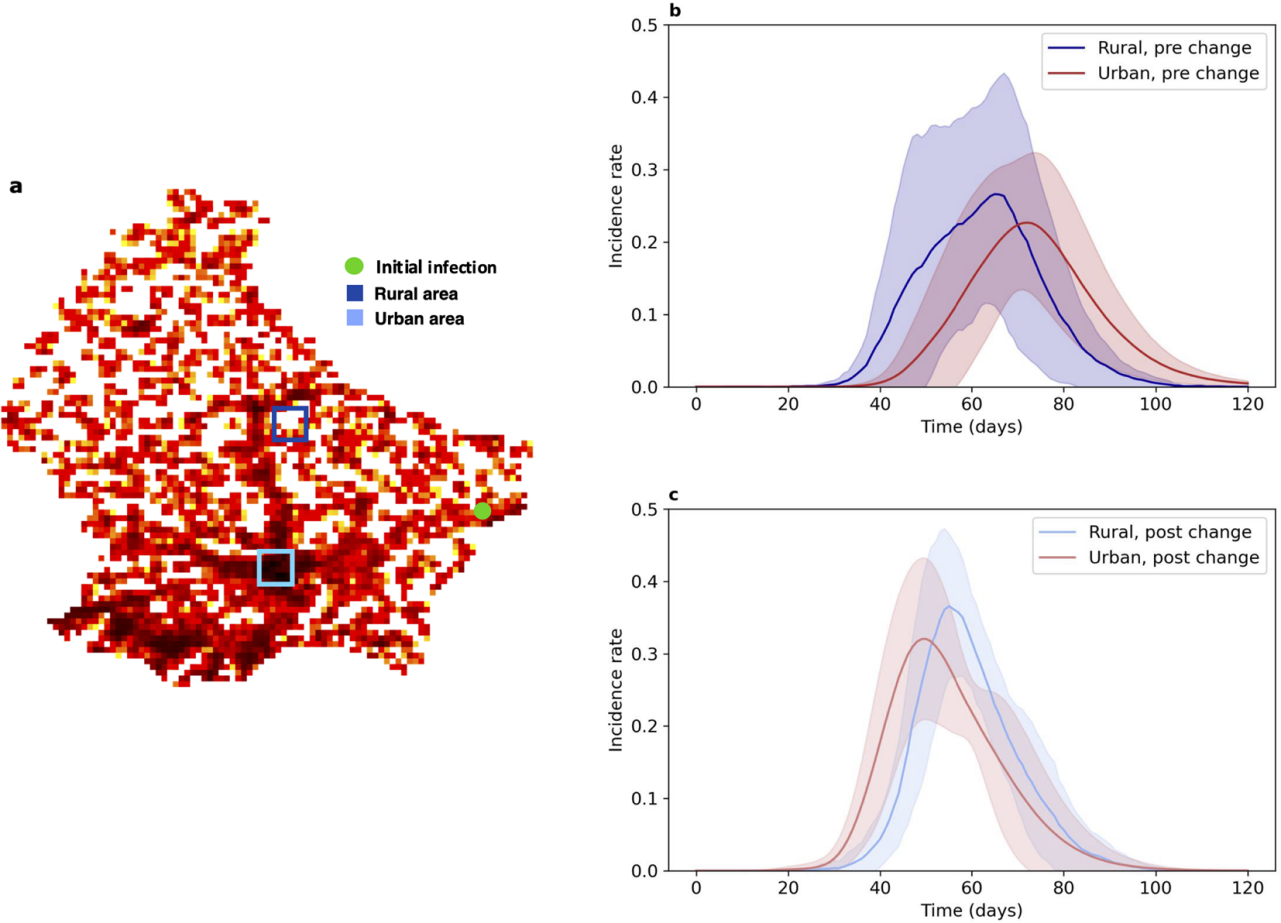
Difference in the spread of disease in Luxembourg in rural and urban populations before and after the change to the spatial weighting. Under the new spatial weighting, the number of infections peaks earlier in the urban region than in the rural region. **(a)** Map highlighting the locations of the urban and rural regions selected for analysis. The urban region is highlighted in light blue and the rural region in dark blue. The location of the initial infections is indicated by the green circle. **(b)** Incidence rate over the course of the simulation for the selected urban (dark red) and rural (dark blue) regions before the change to the spatial weighting. **(c)** Incidence rate over the course of the simulation for the selected urban (light red) and rural (light blue) regions after the change to the spatial weighting. Results are shown as the mean ± standard deviation over 10 repetitions of each simulation. The incidence rate is calculated separately for each region.

As a result of our change to the weighting of the spatial transmission function (so new infections are now guided by both proximity to the infector and population density), the epidemic is now biased towards entering areas of high population density earlier. Comparing the snapshots of the distribution of infections (Fig. 3a and b) with the population density map in Fig. 4a, it can be seen that the distribution of infections at day 40 with the new spatial weighting aligns with the population density distribution. Time-series animations of disease spread before and after the change in the spatial spread of disease are shown in Supplementary Videos 1 and 2. Quantitatively, accounting for population density in spatial transmission results in a substantially shorter and sharper wave of infections, peaking 13 days earlier and at a 32% higher value—that is, an additional 44,180 cases on average (Fig. 3c).

### Epidemic dynamics differ in different density regions

To further elucidate how population density influences disease spread, we compared the shape of the epidemic curve in an urban region, Luxembourg City, with that in the more rural commune of Nommern (Fig. 4a). These locations were selected since they are approximately equidistant from the location selected to seed the initial infections. The areas of the two selected regions were identical, while the population of the urban region was 42,871 compared to 514 in the more rural region.

Under both weightings of spatial transmission, the lower-density population experienced a wave of infections over similar timescales (Fig. 4b and c). However, the higher-density region experienced a much earlier wave of infections after the change to the spatial transmission weightings. Importantly, when spatial infections were guided by distance and population density (under the new spatial weighting), the epidemic enters urban regions earlier, with infections in the urban region peaking earlier than those in the rural region (Fig. 4b). This change in the spatial weighting also resulted in an increase in the average peak number of infections in both the more- and less-densely populated regions.

### Rural and urban environments are similarly impacted by interventions

Based on these differences, we considered whether the impact of non-pharmaceutical interventions differed between higher- and lower-density regions. We applied a combination of interventions to Luxembourg, comprising case isolation and household quarantine for the duration of the 150-day simulation and social distancing from day 49.

**Fig. 5:**
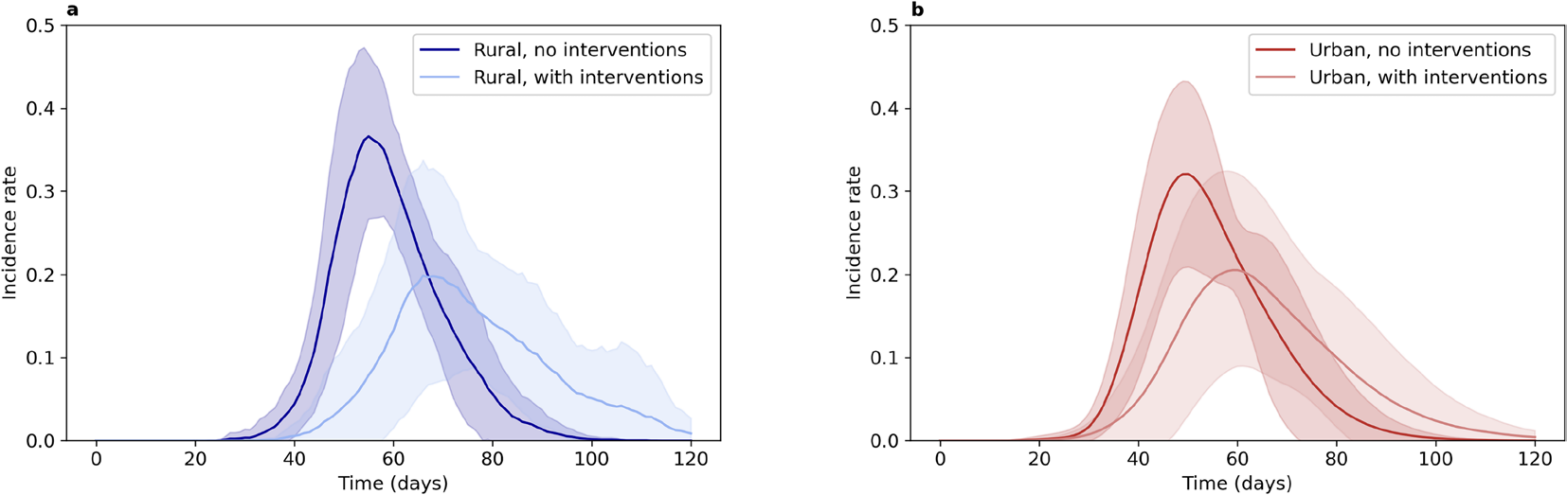
Incidence rate over the course of the simulation for the selected urban and rural regions (of Luxembourg City and Nommern) both with (light red and light blue, respectively) and without (dark red and dark blue, respectively) interventions. Results are shown as the mean ± standard deviation over 10 repetitions of each simulation. The incidence rate is calculated separately for each region.

Comparing the effect of interventions on urban and rural regions, we found that interventions have a similar impact on the urban and rural regions, reducing and delaying the peak of infections in both regions (Fig. 5). Further, both with and without interventions, the incidence rate in the urban region peaks earlier than that in the rural region. However, it should be noted that while the incidence rates show similar patterns, the case numbers in the rural region are much lower than those in the urban region, both with and without interventions. This difference also explains the greater noise in the infection curves for the rural region compared to those for the urban one.

### The impact of interventions may be predicted on a national scale

Finally, to demonstrate how EpiGeoPop and Epiabm can be used together to efficiently construct, run, and visualise epidemic simulations, we constructed a model for the spread of COVID-19 in New Zealand. We compared the effects of different strength interventions to illustrate the range of research that may be conducted with these tools.

We select New Zealand for this case study due to their rapid implementation of a series of strict intervention strategies which successfully controlled the spread of COVID-19 in the early stages of the pandemic^38^. We sought to model the timing of interventions as they were applied in New Zealand between March and May 2020, although we do not undertake detailed parametrisation of the model such that the actual dynamics of COVID-19 are captured. However, we confirm that the reproduction number (*R*) during the initial simulation period is realistic and within the range of COVID-19 variants (see Supplementary Information Section S1.7 for details of this procedure).

We utilised the travel functionality implemented in the new version of Epiabm to introduce one new COVID-19 case to New Zealand each day. The parameter values for the non-pharmaceutical interventions were based on instructions from the New Zealand government as far as possible. Details of the parameters used are provided in the Supplementary Information (Section S1.5).

**Fig. 6:**
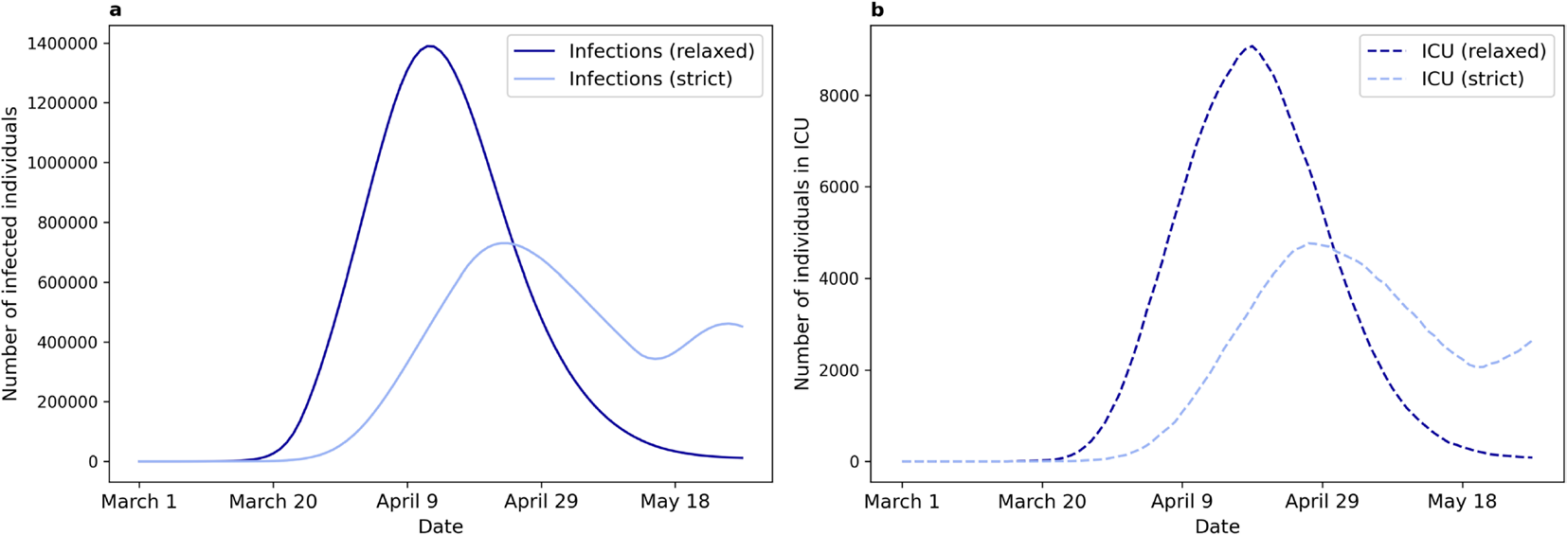
Trajectories of an epidemic in New Zealand with strict and relaxed interventions. The timings of these interventions followed those conducted by the New Zealand government. The effectiveness of case isolation and household quarantine was impaired in the relaxed intervention. **(a)** Number of infected individuals over time under both sets of interventions. **(b)** Number of individuals in intensive care (ICU) over time under both sets of interventions.

We compared the number of infections under strict and relaxed interventions, where the relaxed scenario corresponds to a reduction in the efficacy of case isolation and household quarantine in reducing further infection. The number of infections peaked 12 days earlier and at a 91% higher number of cases in the relaxed scenario compared to the stricter setting, corresponding to approximately 66,100 additional cases at the peak (Fig. 6a). Under the relaxed scenario the number of infections declined more rapidly, while under the more strict interventions a second wave of infections was observed, coinciding with the reopening of schools, workplaces, and outdoor spaces. Similar patterns were observed for the number of patients in intensive care (ICU) over time (Fig. 6b), with a higher and earlier peak observed under the more relaxed interventions compared to the strict interventions, but with a second wave in the latter case. These waves of ICU patients were delayed relative to the wave of infections. Finally, the relaxed scenario led to $\sim 15,000$ more deaths over the course of the simulation than under the strict interventions. Together, these results represent simulations of epidemic-like scenarios in a real country, allowing for qualitative assessments of the impacts of interventions.

## Discussion

Given the role mathematical models played in informing government policy in the recent COVID-19 pandemic, accurate simulation of the spread of disease under hypothetical interventions is crucial for pandemic preparedness. Attention has been paid to the reproducibility of the software used to simulate epidemics, but ensuring a transparent, reproducible origin of the initial model populations is also crucial. Aspects of population heterogeneity, including spatial variation, can be considered within differential equation-based modelling frameworks. For example, age and household structures can be incorporated through additional model compartments^12,14,15^, and meta-population models can be used to capture spatial segregation of subpopulations with individuals travelling between these locations^39^. However, the level of detail achieved with these modelling approaches is generally lower than that represented in ABMs, especially when considering the additional complexity often present in ABMs due to the simultaneous incorporation of multiple types of heterogeneity.

Here, we present EpiGeoPop, a Python workflow for generating spatially accurate population input files based on real-world population density data, facilitating the application of ABMs to real-world countries and practical research questions. This interfaces with Epiabm^33^, an alternative implementation of CovidSim^4^, which prioritises good software engineering practices and which we extended to simulate a range of pandemic interventions.

Taken together, EpiGeoPop and Epiabm allow for simulation of populations with real-world age, household, and spatial distributions. EpiGeoPop reads in and processes spatial population density data, preserving exact spatial distributions, and we have verified that the functions within Epiabm used to generate realistic household configurations perform this task with high fidelity, reproducing the age and household distributions used as input parameters (Fig. S1). EpiGeoPop uses the comprehensive population density dataset provided in the Global Human Settlement Layer^34^ to configure model populations for any country of interest. While a critical evaluation of different available datasets is beyond the scope of this paper, it is important to acknowledge that the accuracy of EpiGeoPop outputs is contingent on the accuracy of the underlying population density data, and, in certain regions around the world, knowledge of the true population density may be subject to substantial uncertainty^40^.

While EpiGeoPop preserves certain demographic features, several aspects of real-world population complexity are not captured in our synthetic populations. Importantly, the model does not incorporate detailed mobility patterns such as commuting flows between activity spaces (e.g., schools and workplaces). This represents a key limitation of the model, since in reality contact patterns extend beyond geographic proximity, which is the basis of the model’s spatial transmission pathways. Although place-based transmission is included in the model, difficulties accessing comprehensive yet tractable data on place locations, capacities, and usage patterns means there is limited scope at present to develop tools that utilise this information. Additionally, our approach assumes that household composition and age distributions are uniform across each country, whereas in real populations these demographic characteristics may vary substantially between different regions within a country. Relevant to our comparison of urban and rural regions, previous analyses have shown that age distributions of rural populations skew towards older, retired age groups, while urban populations include more working-age individuals^41^. By assuming age distributions that are uniform across each country, we are not able to replicate these distinct patterns (Fig. S2). Although such regional-level data is not available globally, extending EpiGeoPop to allow for different age and household distributions to be applied to different geographic regions where they are accessible would be an interesting avenue for future work.

Combining EpiGeoPop with the updated version of Epiabm (v1.1.0), we demonstrated the importance of including spatial detail in epidemiological models, showing how incidence rates may differ according to regional density and the impact of interventions on high-density regions compared to lower-density ones. In particular, our change to the weighting of the spatial transmission function resulted in the epidemic entering higher-density regions earlier.

This result is in line with observations made during the COVID-19 pandemic that infection numbers often peaked in urban regions before rural regions^42,43^. We propose that weighting spatial transmission according to both distance and population size better captures this pattern of infections being focused on large cities during the early phases of an epidemic since we better approximate spatial contact networks. This change assumes that individuals are more likely to visit an area with a larger population than an area with a smaller population, given the two are the same distance away. This assumption is reflected in studies of complex spatial contact networks, such as work by Giles et al.^44^, who analysed mobile phone data from Namibia to investigate commuting patterns, identifying that the highest volume of trips were short-duration trips between high-density districts. However, as discussed above, the full complexity of individual mobility patterns described by these spatial contact networks are not captured with our current approach.

Our results provide a basis for future work investigating how spatial patterns influence the spread of disease and the response to intervention. A number of recent studies have sought to elucidate the impact of population density on the spread of COVID-19, reaching contradictory conclusions regarding whether COVID-19 spread was affected by^45–47^ or independent of^27,48^ population density. Since previous studies have been retrospective in nature, looking for associations between population density and the number of infections, hospitalisations, and deaths, the results are heavily influenced by the interventions in place in these regions and other demographic and socioeconomic characteristics^49^. In order to gain insight into the role of population density to aid preparation for future pandemics, predictive studies modelling the impact of a range of interventions in specific geospatial contexts are essential.

Previously, Hamidi et al.^27^ suggested that although higher-density regions might experience earlier and perhaps higher waves of infections, better health systems in such places may explain why these regions experience lower numbers of deaths overall when compared to lower-density areas. This importance of healthcare provision on the overall impact of an epidemic is reflected in the focus of policy-driven modelling on predicting required hospital and ICU capacity. In this work, we presented simulations comparing the number of patients requiring ICU treatment in New Zealand under strict and more relaxed interventions. Under both sets of interventions, the number of ICU patients would have greatly exceeded the country’s capacity of around 200 beds^50^. While in reality New Zealand pursued a zero-COVID strategy, simulations such as those presented here could be used in future pandemic scenarios to identify the level of restrictions needed to ensure healthcare capacity is not overwhelmed. Similar simulations could be conducted to specifically compare outcomes between rural and urban regions, connecting prospective simulation work with the observations from retrospective studies^27,43^.

As more comprehensive datasets become available, EpiGeoPop and Epiabm could also be leveraged to capture additional population heterogeneities. While the age, household, and spatial complexity that can be achieved by combining EpiGeoPop with Epiabm represents an advancement on previous models, the inclusion of further heterogeneities would enhance the real-world applicability of these models. Several aspects of population heterogeneity not considered here have been considered in the field of synthetic population generation, including ethnicity, occupation, and day-to-day mobility patterns^51–53^. However, the use of census data to generate demographically realistic populations is a time-consuming exercise requiring a degree of expertise in census data. This significant investment of time and effort results in highly country-specific populations tailored to the research question of interest. There is therefore a need to develop more flexible and automated approaches to population generation to facilitate simulation studies. By streamlining the inclusion of data where it is currently readily accessible, we hope that EpiGeoPop may enable researchers to develop spatially realistic disease simulations representing their country of interest.

In summary, EpiGeoPop represents a flexible tool for generating spatially accurate input files for investigating the spread of disease, which can be built upon to be of continued use to the field. By automating population configuration, EpiGeoPop streamlines the process of obtaining and formatting the large amounts of information needed by ABMs, and we hope that its public availability will support standardisation of input formats within agent-based epidemiological modelling.

## Supplementary Information

Supplementary Information 1.

Supplementary Information 2.

Supplementary Information 3.

## Data Availability

PyEpiabm (v1.1.0)^54^ is available from https://doi.org/10.5281/zenodo.10016636. EpiGeoPop (v1.0.0)^55^ is available from https://doi.org/10.5281/zenodo.14112521. EpiGeoPop uses population density data from https://jeodpp.jrc.ec.europa.eu/ftp/jrc-opendata/GHSL/GHS_POP_MT_GLOBE_R2019A/GHS_POP_E2015_GLOBE_R2019A_4326_30ss/V1-0/, regional boundaries from https://www.naturalearthdata.com/downloads/10m-cultural-vectors/, and age distribution data from https://population.un.org/wpp/Download/SpecialAggregates/EconomicTrading/. Details regarding the Luxembourg simulations can be found at https://github.com/SABS-R3-Epidemiology/epiabm/tree/main/python_examples/luxembourg_example. Details regarding the New Zealand simulations can be found at https://github.com/SABS-R3-Epidemiology/epiabm/tree/main/python_examples/NZ_example. Information on the timing of restrictions in New Zealand was found at https://covid19.govt.nz/about-our-covid-19-response/history-of-the-covid-19-alert-system/
