# Supplementary figures and images for: EpiGeoPop: a tool for developing spatially accurate country-level epidemiological models
Source: Sci Rep. 2025 Jul 22;15:26663. doi: 10.1038/s41598-025-11999-4 (PMC12284068; doi:10.1038/s41598-025-11999-4)

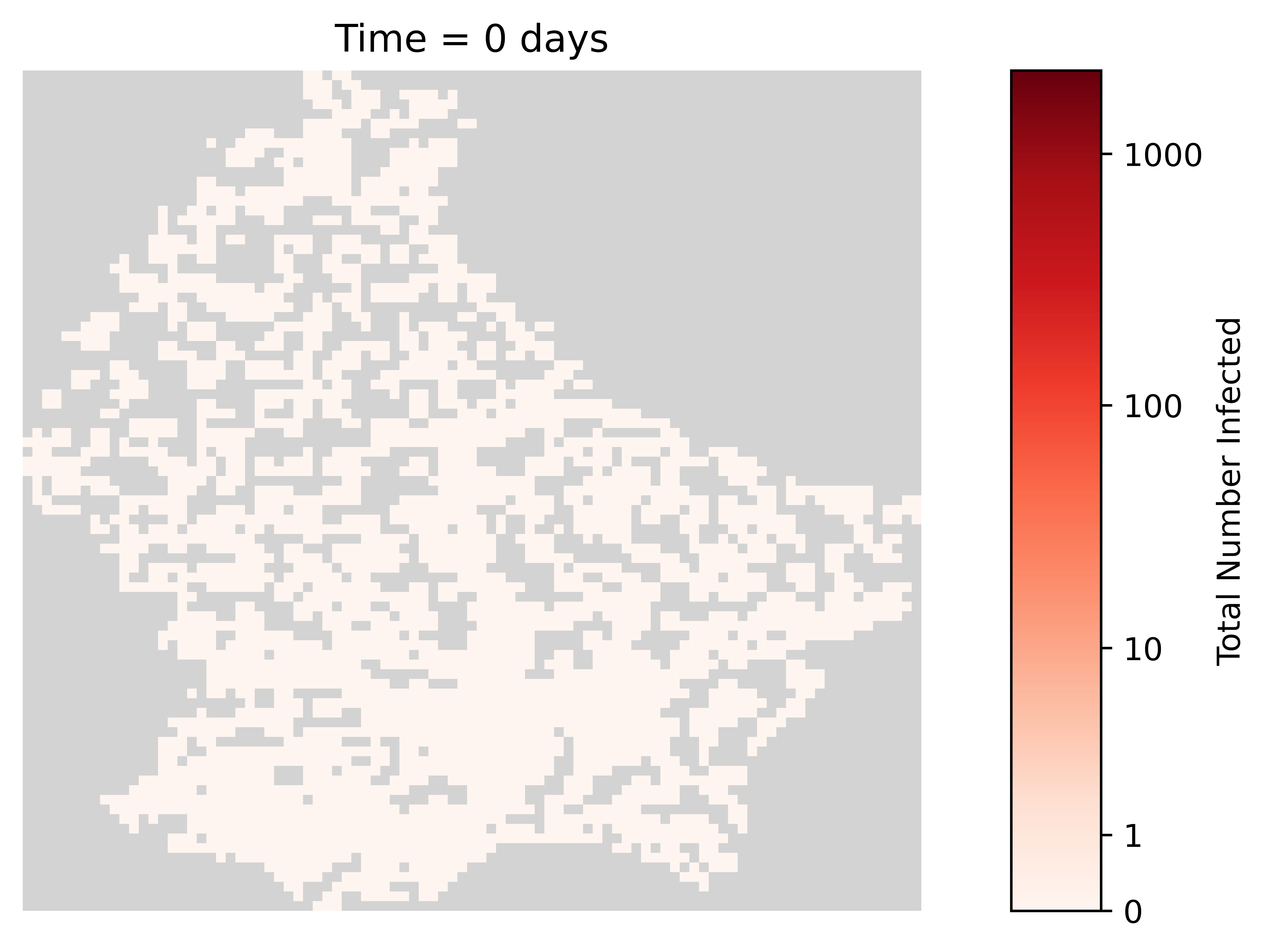

Supplement: Supplementary file 1 — Supplementary Information 1. [file 41598_2025_11999_MOESM1_ESM.gif]

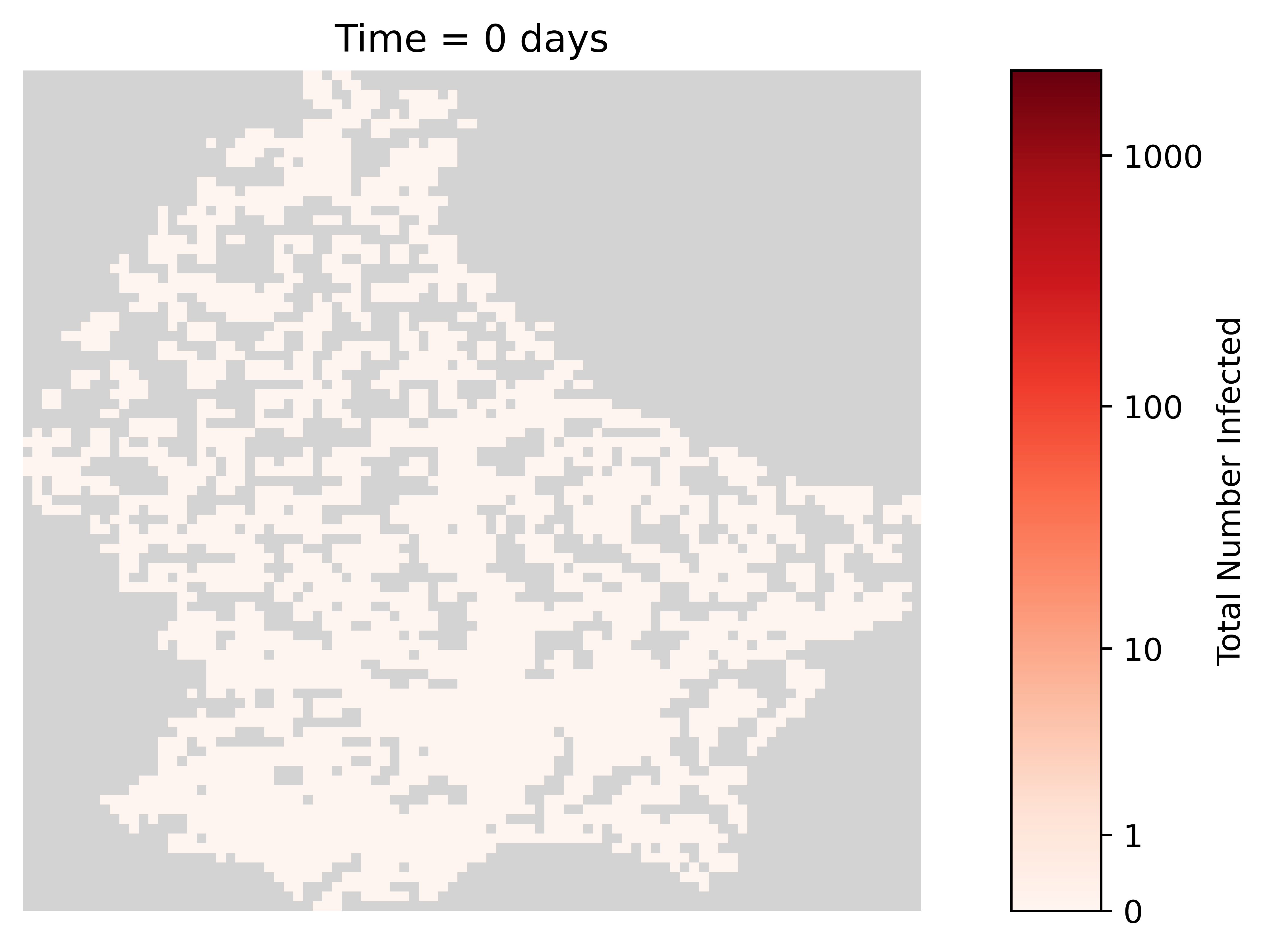

Supplement: Supplementary file 2 — Supplementary Information 2. [file 41598_2025_11999_MOESM2_ESM.gif]
