# Supplementary material for: EpiGeoPop: a tool for developing spatially accurate country-level epidemiological models
Source: Sci Rep. 2025 Jul 22;15:26663. doi: 10.1038/s41598-025-11999-4 (PMC12284068; doi:10.1038/s41598-025-11999-4)
Supplement: Supplementary file 3 — Supplementary Information 3. [file 41598_2025_11999_MOESM3_ESM.pdf]

Supplementary Information for EpiGeoPop: A Tool for Developing Spatially Accurate Country-level Epidemiological Models

Lara Herriott[1,+], Henriette L. Capel[1,+], Isaac Ellmen[1,+], Nathan Schofield[1,+], Jiayuan Zhu[1,+], Ben Lambert[4], David Gavaghan[1,3], Ioana Bouros[1, 3], Richard Creswell[3], and Kit Gallagher[1,2]

[1]SABS R³ CDT, University of Oxford, UK
[2]Mathematical Institute, University of Oxford, UK
[3]Department of Computer Science, University of Oxford, UK
[4]Department of Statistics, University of Oxford, UK
[*]gallagher@maths.ox.ac.uk
[+]these authors contributed equally to this work

Supplementary Information

S1.1 EpiGeoPop usage

Details on how to install and run the EpiGeoPop workflow are provided at https://github.com/SABS-R3-Epidemiology/EpiGeoPop?tab=readme-ov-file#running. The example provided will generate the relevant output files for Luxembourg, comprising:

1. Population density map (e.g., outputs/Countries/Luxembourg.pdf),
2. Input file for the epidemiological simulation (e.g., data/processed/countries/Luxembourg_microcells.csv),
3. Json file containing age distributions for the population of interest (e.g., data/processed/countries/Luxembourg_pop_dist.json).

Running the Snakemake pipeline for Luxembourg on an Apple M2 Pro (8 core CPU) took 5.8s, demonstrating the efficiency of this tool for generating spatially accurate population input files, a task which previously represented a substantial bottleneck in the utility of ABMs.

The prep.sh file downloads the relevant data in advance of running the Snakemake pipeline. As such, by changing the web address of the data sets, updated population density data can easily be used as long as the format matches that of the data currently used. The prep.sh file provided in EpiGeoPop downloads spatial data at a resolution of 30 arc seconds.

To run the workflow for a different geographic region, the user should first create a configuration file (labelled as name_paramerers.json) in the relevant folder within the configs directory. This configuration file should take the same format as those provided, and can define parameters including the number of microcells each cell should be broken into, the average number of places to assign per microcell, the household size distribution for the region of interest, and, crucially, the name of the region of interest. Then, the user should update Snakefile to refer to their geographic region of interest, as indicated by commented-out examples.

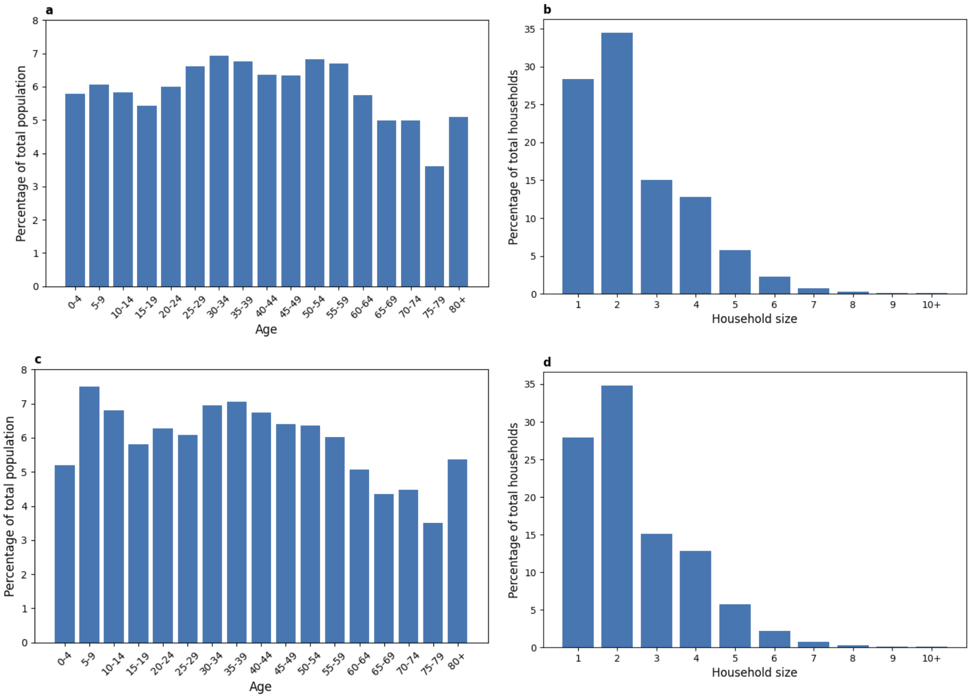

Figure S1. Comparisons of the input and output population age and household distributions. **(a)** Age distribution for the UK, used as an input parameter. **(b)** Household size distribution for the UK, used as an input parameter. **(c)** Age distribution obtained after applying population configuration algorithm. **(d)** Household size distribution obtained after applying population configuration algorithm.

S1.2 Validation of the age and household assignment in Epiabm

We validated that the algorithm used to assign individuals to households, and subsequently assign ages within households, generated realistic household structures that reflected the desired age and household size distributions.

To do this, we run the initial_household_sweep function from Epiabm over an example population of 10,000 individuals using parameters based on the UK population. The desired age and household size distributions, which are provided as input parameters, are shown in Figure S1a and b, respectively. After running the age and household assignment algorithm, we obtained a model population with overall age and household size distributions (Figure S1c and d, respectively) that were similar to the desired distributions. Although there are some differences between the desired and obtained age distributions, the overall shape of the distribution is similar. The differences that are observed can be attributed to the large number of parameters relevant to household composition that are required by the algorithm (such as the average difference in age between two children in the same household). We use a parameter set used for the UK in the original CovidSim simulations.

S1.3 The impact of non-pharmaceutical interventions

As an example, we demonstrate the impact of stacking interventions on a model population of 10,000 individuals spread uniformly over a 4x4 grid, beginning with case isolation and then adding household quarantine and subsequently place closure (Figure S3). The interventions decrease and delay the height of the peak of infections, with place closure having by far the strongest effect under the parameters chosen. Moreover, place closure substantially decreases the total number of infections. The relative impact of each intervention is highly dependent on the context of the population and characteristics of the population in question. As such, the greater impact of place closure compared to other interventions in this example will be influenced by the number of places and households per person in the model population. The parameters used for this simulation can be found at https://github.com/SABS-R3-Epidemiology/epiabm/blob/main/python_examples/model_population_example/IntMult_params.json.

S1.4 Luxembourg simulation details

For the initial conditions used in the Luxembourg simulations presented in Figures 3, 4, and 5, we seeded five initial infections in the same cell (highlighted in Figure 4) towards the furthest eastern part of Luxembourg near the border with Germany $(6.400^\circ N, 49.708^\circ W)$. The urban and rural locations selected, Luxembourg city and Nommern, respectively, are both $\sim 180km$ from this initial infection site as the crow flies, allowing for fair comparisons to be made. Simulations were repeated 10 times and results are presented as mean ± standard deviation.

While the model population densities of Nommern and Luxembourg city differ as expected, some specific variations in demographic patterns between these regions are not captured. For example, the population of Nommern includes a higher proportion of older individuals than that of Luxembourg city. However, since we apply a single country-level age distribution when configuring the population, our model population does not reflect these differences (Figure S2). The variation in age distributions between regions that is observed in the model population is the result of random noise in our age and household assignment algorithms.

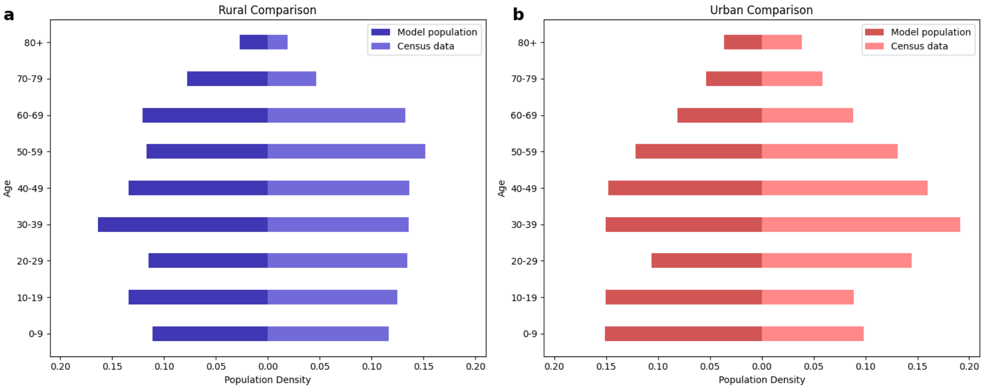

Figure S2. Comparisons of the configured age distributions from selected regions in the model population, against the corresponding census data[1]. **(a)** Age distributions for Nommern, our selected rural region. **(b)** Age distributions for Luxembourg city, our selected urban region.

For the simulations with interventions, we applied case isolation and household quarantine for the first 90 days and social distancing between days $49-90$. The parameters governing the simulations can be found at https://github.com/SABS-R3-Epidemiology/epiabm/tree/main/python_examples/luxembourg_example: for simulations without interventions see luxembourg_parameters.json and for simulations with interventions see luxembourg_intervention_parameters.json.

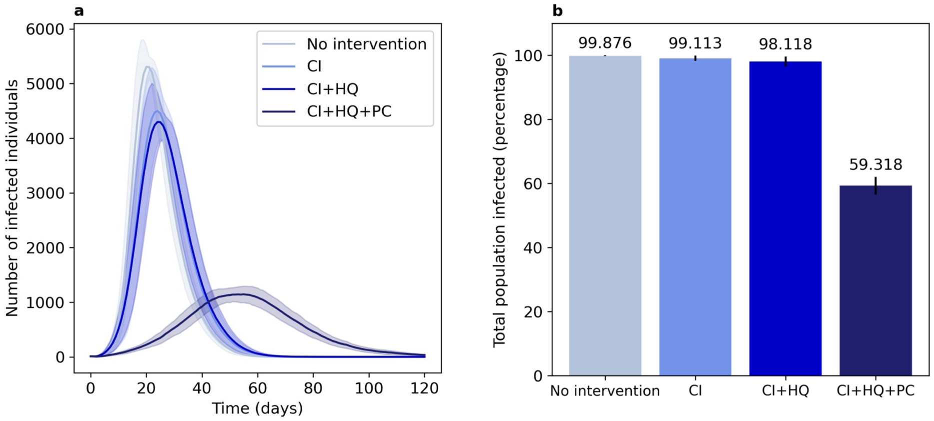

Figure S3. Comparison of disease transmission between different number of active interventions. (**a**) Shows the number of infected individuals over time. (**b**) Shows the total number of infected individuals over the entire simulation period. Simulations are run on a population of 10,000 individuals spread approximately uniform over a squared 4x4 grid. Without an intervention (grey) 97.85 ± 0.16 percent of the population will get infected with the highest peak of 5453 infected individuals at day 20. The Case Isolation (CI, light blue) intervention showed similar number of infections (96.69 ± 0.66 percent) but the peak of the wave is reduced to 4600 and delayed to day 24. The Household Quarantine (HQ) intervention as an additional intervention on top of CI (blue, 95.67 ± 1.23 percent) slightly decreased the height of the infectious peak to 4408 at day 24. The total number of infections (56.94 ± 3.38 percent), height of the peak (1089), and the day at which this peak is observed (day 48) are substantial decreased and delayed by introducing the Place Closure (PC) intervention as additional intervention (dark blue).

S1.5 New Zealand simulation details

In the New Zealand simulation, the parameter values for non-pharmaceutical interventions were based on instructions from the New Zealand government as far as possible. According to the timeline of Alert Level changes, which documents the dates of key events and the duration of the State of National Emergency (https://covid19.govt.nz/about-our-covid-19-response/history-of-the-covid-19-alert-system/), we extract the following parameters to use in our simulation of strict intervention.

Each simulation starts on 1st March and lasts for 90 days. From 14th March, international arrivals are required to quarantine in a hotel for two weeks. Non-traveller individuals must self-isolate for two weeks if they are symptomatic for the duration of the simulation. Members of the household of symptomatic individuals are subject to household quarantine for two weeks. Household quarantine is set to have a 75% compliance rate while that of case isolation is 100%. In addition, all schools, workplaces and outdoor spaces are closed from day 25 to day 74. Social distancing is conducted from day 49 to the end of the simulation.

Simulations are run on high-performance computing facilities provided by the University of Oxford. Simulations are run on one compute node with 256 GB CPU memory. Due to limited access to compute resources, only one repetition was run to provide an qualitative insight into the impact of interventions.

The parameters governing the simulations can be found at https://github.com/SABS-R3-Epidemiology/epiabm/tree/main/python_examples/NZ_example: for simulations with strict interventions see NewZealand_parameters.json and for simulations with more relaxed interventions see NewZealand_parameters_relaxed.json.

S1.6 The balance between household, place, and spatial infections

The basic reproduction number (R_0) in ABMs, and models with multiple levels of mixing more generally, may be challenging to compute as it emerges from the interaction of multiple different transmission mechanisms. In Epiabm the infectiousness of the virus is governed by three parameters, 'spatial_transmission', 'household_transmission', and 'place_transmission', which act on top of the defined infectiousness profile to control the number of individuals infected at each time step through spatial, household, and place infection pathways, respectively. Translating these parameters to an effective R_0 is non-trivial, and a range of different approaches have been implemented in the literature[2–4]. For example, CovidSim scales the number of spatial infections by the parameter 'local_beta', which is calculated by considering the number of spatial infections which are needed on top of place and household infections to obtain the target R_0 selected by the user as an input parameter.

We studied the influence of the spatial infectiousness parameter (i.e., the parameter governing the number of individuals selected to be infected via the spatial transmission pathway by a given infectee) on the infection events that take place

in households, places, and spatially by infecting one person in an entirely susceptible population. The code used for this investigation can be found at https://github.com/SABS-R3-Epidemiology/epiabm/tree/study-r0/python_examples/r0_testing_simulation. The number of infections caused by this initial infection over a period of 21 days is studied for different spatial infectiousness parameter values (Figure S4). Since we do not count secondary infections, these investigations provided a measure of R_0 as traditionally defined. Since the spatial infectiousness parameter does not apply to the household and place transmission pathways, only spatially derived infections scale with the spatial infectiousness parameter with a slope of 0.42. As such, the total number of infections scales in the same way as spatial infections alone, but shifted to a higher value according to the number of household and place infections.

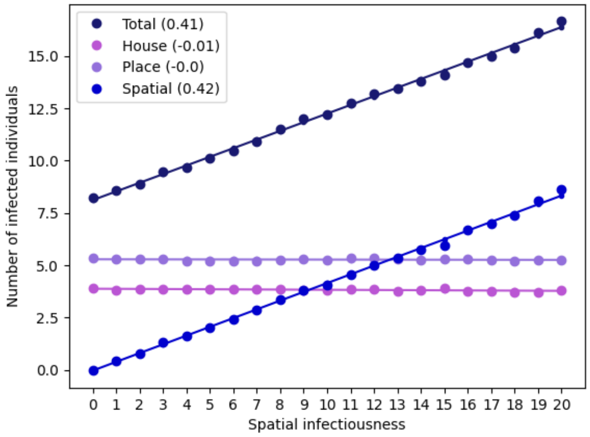

Figure S4. Household, place, spatial, and total infections for varying spatial infectiousness parameter values. The spatial infectiousness parameter governs the number of individuals selected to be infected via the spatial transmission pathway by a given infectee. Simulations were run on a population of 10,000 uniformly distributed over 200 cells for 21 days following a single initial infection. The infections events in their household (purple), the places they visit (light blue), spatially (blue), and the total infections (dark blue) of this single individual over a period of 21 days are shown. Linear curves were fitted and slopes are indicated between brackets in the figure labels. Spatial infections scale linearly with the spatial infectiousness parameter with a slope of 0.42.

The 'spatial_infectiousness' parameter here, and the 'local_beta' parameter in CovidSim, only scale spatial infections. However, these spatial infections include all infections between cells, and are thus intended to capture a proportion of infections in places and in visits to other households. The concept of place infections in particular therefore needs to be more cautiously interpreted as only representing infections through places within the person's immediate local area (their cell). Furthermore, Figure S4 shows that place infections dominate over the other transmission pathways for spatial infection values below 12, given the parameterisation used. However, a number of previously published analyses based on contact tracing data and genomic surveillance[5,6] indicate that household infections dominate.

Addressing the substantial challenges associated with implementing R_0 in agent-based models is beyond the scope of this paper. To ensure our simulations fall within the reasonable range for R_0, we use an approximate value for 'spatial_transmission' which results in an initial time-dependent reproduction number (R_t) of approximately 3 across all simulations. R_t is the expected number of infections generated by someone infected at time t over the course of their infectious period[7,8]. We determine this by running inference on the infection curves for each result to calculate a rolling estimate of R_t[7].

S1.7 Inference of R_t using branchpro

We use branchpro, an inference tool based on branching process models, to infer the R_t profile over the first 30 days under both the strict and relaxed interventions in New Zealand (Figure S5). We use serial intervals tailored to COVID-19 and the following parameters for the inference: τ, the rolling window over which R_t is averaged, was set at 7 days; ε was set to 1, meaning the transmission risk of an imported case is deemed equal to local ones; and α and β, the shape and rate parameters of the gamma prior, were set to 1 and 0.2, respectively.

We take the mean inferred R_t over the first 7 days as an estimate of R_0 over each simulation. Under the strict interventions we obtain an estimate of R_0 of 3.55 ± 1.02, while under the more relaxed interventions we estimate R_0 to be 5.16 ± 1.62.

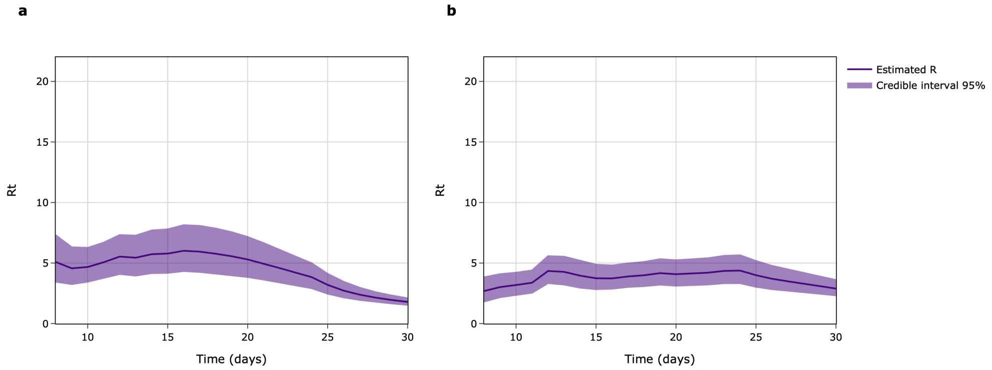

Figure S5. Inference of R_t under the strict and more relaxed interventions in New Zealand. **(a)** R_t profile for the relaxed interventions scenario. **(b)** R_t profile for the strict interventions scenario. In both plots the solid purple line indicates the mean estimate of R_t and the shaded purple area represents the 95% central credible interval of the R_t posterior.

References

1. STATEC. Population by canton and municipality, sex and age. Available from: https://lustat.statec.lu/?lc=en&pg=0&ac=false.
2. Ferguson, N. M. *et al.* Strategies for mitigating an influenza pandemic. *Nature* **442**, 448–452, DOI: 10.1038/nature04795 (2006).
3. Hunter, E., Namee, B. M. & Kelleher, J. An open-data-driven agent-based model to simulate infectious disease outbreaks. *PLOS ONE* **13**, e0208775, DOI: 10.1371/journal.pone.0208775 (2018).
4. Pellis, L., Ball, F. & Trapman, P. Reproduction numbers for epidemic models with households and other social structures. I. definition and calculation of R0. *Math. Biosci.* **235**, 85–97, DOI: https://doi.org/10.1016/j.mbs.2011.10.009 (2012).
5. Anderson, R., Vegvari, C. & Baggaley, R. SARS-CoV-2: Where do people acquire infection and 'who infects whom'? *The Royal Soc.* (2020).
6. Thompson, H. A. *et al.* Severe acute respiratory syndrome coronavirus 2 (SARS-CoV-2) setting-specific transmission rates: A systematic review and meta-analysis. *Clin. Infect. Dis.* **73**, e754–e764, DOI: 10.1093/cid/ciab100 (2021).
7. Creswell, R. *et al.* Heterogeneity in the onwards transmission risk between local and imported cases affects practical estimates of the time-dependent reproduction number. *Philos. Transactions Royal Soc. A: Math. Phys. Eng. Sci.* **380**, 20210308, DOI: 10.1098/rsta.2021.0308 (2022).
8. Thompson, R. N. *et al.* Key questions for modelling COVID-19 exit strategies. *Proc. Royal Soc. B: Biol. Sci.* **287**, 20201405, DOI: 10.1098/rspb.2020.1405 (2020).
